# Fluoride Release from Two High-Viscosity Glass Ionomers after Exposure to Fluoride Slurry and Varnish

**DOI:** 10.3390/ma12223760

**Published:** 2019-11-15

**Authors:** Hani M. Nassar, Jeffrey A. Platt

**Affiliations:** 1 Department of Restorative Dentistry, Faculty of Dentistry, King Abdulaziz University, P.O. Box 80209, Jeddah 21589, Saudi Arabia; 2 Department of Biomedical Sciences and Comprehensive Care, School of Dentistry, Indiana University, 1121 West Michigan Street, Indianapolis, IN 46202, USA; jplatt2@iu.edu

**Keywords:** fluoride release, glass ionomer cements, toothbrushing, abrasion

## Abstract

The effect of brushing with different fluoride slurries on the fluoride release (FR) of different high-viscosity glass ionomer cements (GICs) was investigated. Fifty-eight discs were fabricated from two high-viscosity GICs (GC Fuji IX (F9) and 3M ESPE Ketac-fil (KF)). Five specimens from each brand were used to measure Vickers microhardness and the remaining were randomly assigned to one of four groups (*n* = 6) based on two-factor combinations: (1) fluoride concentration in the abrasive slurry (275 or 1250 ppm fluoride as NaF) and (2) immersion in a 22,500 ppm fluoride-containing solution. Specimens were brushed for a total of 20,000 strokes over 4 days with daily FR measurement. Data were analyzed using analysis of variance and Bonferroni tests (α = 0.05). Baseline FR and microhardness values were different between the two tested material brands. Exposure to a 22,500 ppm solution was associated with higher FR but not the exposure to 1250 ppm slurries. Brushing and immersion of glass ionomer cements in a 22,500 ppm F solution led to higher FR that was more sustained for KF. Type of the glass ionomer, progressive brushing, and fluoride varnish affected FR but not the fluoride content in the abrasive slurry.

## 1. Introduction

Fluoride release from high-viscosity glass ionomer cements (GICs) has been proposed as a possible mechanism for reducing the incidence of dental caries [1,2]. Multiple reports have shown that there is a large initial dose of fluoride release when GIC is placed intraorally which tapers down after 48 h reaching a prolonged steady level [3,4,5,6,7,8]. Consequently, augmenting this initial release is advisable in order to maintain adequate levels to hinder the caries process. Daily exposure to fluoride-containing toothpastes can stimulate fluoride recharging and subsequent release from GIC [8,9,10]. Furthermore, it was reported previously that fluoride release from GIC can be enhanced when specimens are brushed in the presence of a fluoridated dentifrice slurry [11]. Additionally, brushing with slurries containing high abrasive loads increases the fluoride release from conventional GICs [12]. Still, fluoride release can be dependent on other factors, such as the fluoride concentration of the slurry as well as the brand of the material. Brushing with prescription toothpaste containing 5000 ppm fluoride and using a fluoridated mouth rinse are two recommendations that clinicians give to their patients to manage and prevent dental caries according to established caries management protocols such as CAMBRA and Cariogram [13,14,15]. These factors would allow the exposure of GICs to higher fluoride concentrations potentially leading to an enhancement of fluoride recharge and release.

The objective of this study was to investigate the effects of slurry fluoride concentration and material brand on the fluoride release from conventional glass ionomer cements in the presence of a fluoride varnish-simulated step. We hypothesized that brushing with higher fluoride concentration and the presence of a simulated varnish step would enhance the fluoride release from high viscosity glass ionomer material depending on the tested brand.

## 2. Materials and Methods

### 2.1. Specimen Preparation and Group Allocation

A 2 × 2 × 2 factorial design was used from the interaction of three factors: glass ionomer brand (GC Fuji IX, GC America, Alsip, IL, USA (F9) and 3M ESPE Ketac-fil Plus Aplicap, 3M, St. Paul, MN, USA (KF)), fluoride concentrations in the slurry (275 and 1250 ppm fluoride as NaF), and inclusion of intermediary exposure to a fluoride varnish step (yes, no). Twenty-nine specimens (shade: A2, dimensions: 6.2 mm diameter and 3.0 mm thickness) were made from each glass ionomer brand. Following manufacturers’ instructions, the GIC capsule was activated and mixed in an amalgamator for 10 s. GIC was manipulated into a stainless steel mold on top of a glass slab in one increment and covered with a Mylar strip. A glass slide was used to press the material in order to make it flush with the margins of the mold before allowing the specimens to self-cure. After 3 min, the specimen was removed from the mold and was placed in a humidor at 37 °C after marking one side of the specimen.

### 2.2. Fluoride Release and Specimen Allocation

Each GIC disc was individually immersed in a fluoride-free plastic vial containing 5 mL of deionized (DI) water for 24 h. Baseline fluoride release passively diffused during the storage period was measured afterwards by pipetting 1 mL of the storage medium into a new vial and mixing it with 1 mL of a total ionic strength adjustment buffer II solution (R8670000; Ricca Chemical Company, Arlington, TX, USA). Fluoride release was indirectly calculated by measuring the millivoltage of the sample using a fluoride electrode (Orion Research, Inc., Boston, MA, USA) connected to an ion analyzer and compared with a standard curve. Baseline fluoride release was used to allocate 24 discs from each material, following a stratified randomization approach, to either brushing with slurries containing 275 ppm fluoride (content in regular toothpaste after a 1:3 dilution) or 1250 ppm fluoride as NaF (fluoride content in prescription toothpaste after a 1:3 dilution). Half of the specimens were exposed to a solution containing 22,500 ppm fluoride representing a varnish step resulting in 4 groups (*n* = 6) per GIC brand for a total of 24 specimens. Additional fluoride release measurements were done daily after overnight immersion in fresh DI water and also after 7 days post-brushing.

### 2.3. Brushing and Treatment Protocol

After specimen allocation, specimens were placed in custom-made resin blocks and were brushed for 5000 strokes (150 g load) simulating approximately 30 min of continuous brushing with the assigned slurry (Relative Enamel Abrasivity (REA) = 4.0) and stored in fluoride-free vials containing 5 mL of deionized water (DI) for 24 h. Discs from groups to be exposed to the fluoride varnish-mimicking step were immersed in a solution containing 22,500 ppm fluoride as NaF for 1 h before being placed in new fluoride-free vials containing 5 mL of DI for 24 h (Table 1). Each assigned protocol was repeated for 4 days with daily fluoride release measurement for a total of 20,000 strokes at the end of the period. Specimens were stored in DI for 7 additional days without brushing and fluoride release for the seventh day was measured and reported.

### 2.4. Microhardness Testing

Five specimens from each brand were used for measuring Vickers microhardness (Leco Microhardness Tester, model M-400, Leco Co., Tokyo, Japan) after immersing the specimen for 24 h. A 1 N load was applied for 10 s and an average of 3 measurements was reported and used to determine the Vickerss Hardness Number (VHN) for each disc.

### 2.5. Statistical Analysis

A mixed repeated measure analysis of variance (ANOVA) was utilized to determine the effects of material type, slurry fluoride level, presence of a varnish step, and time effect along with their interactions. To determine changes of fluoride release within each group across different time points, One-way ANOVA was used. Pairwise comparisons were done using the Bonferroni method. Two-sample t-tests were used to compare baseline fluoride release as well as microhardness values between the two material types. All statistical analyses were conducted at 5% significance level using SPSS Statistics 17.0 (IBM Corporation, Armonk, NY, USA).

## 3. Results

There was a significant difference between the two materials regarding microhardness with KF (VHN = 32.6 ± 11.3) showing significantly higher values (*p* = 0.024) compared to F9 (VHN = 14.7 ± 4.9). Also, significantly higher (*p* = 0.02) fluoride release values were recorded for KF (47.7 ± 12.0 µg/mL) compared to F9 (37.0 ± 14.2 µg/mL).

There was a significant effect for material type (*p* < 0.001) and varnish step (*p* < 0.001) with KF associated with more fluoride release overall and added effect in the varnish-exposed groups except for baseline and one week post-brushing (Table 2). Slurries with 1250 ppm did not show a significant increase in fluoride release.

**Figure 1:**
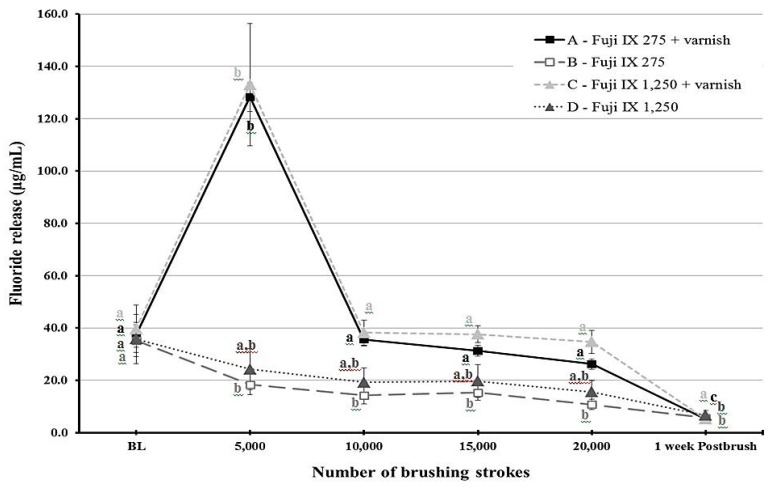
A line graph showing the mean fluoride release from Fuji IX (F9) material at different time points throughout the study period. Error bars indicate standard deviation. Same letters indicate non-significant differences (*p* > 0.05) between values at different brushing levels within the same group.

**Figure 2:**
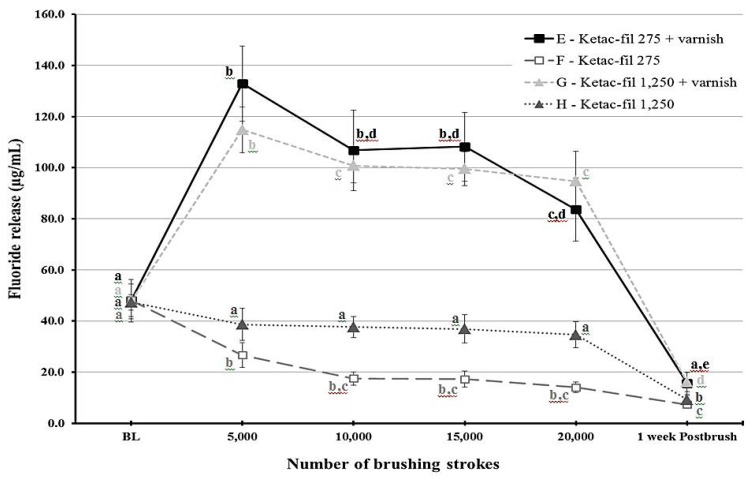
A line graph showing the mean fluoride release from Ketac-fil (KF) material at different time points throughout the study period. Error bars indicate standard deviation. Same letters indicate non-significant differences (*p* > 0.05) between values at different brushing levels within the same group.

Fluoride release was different for each time point depending on the group (*p* < 0.001). Figure 1 and Figure 2 show statistical comparisons for F9 and KF groups over time, respectively. The highest fluoride release values were recorded at 5000 strokes in the varnish groups of each material. For F9, fluoride release values sharply decreased after 10,000 strokes for the varnish groups (groups A and C) and remained low throughout the study period for the other two groups (B and D). Regarding KF, the varnish groups (E and G) released more fluoride compared to the non-varnish groups (F and H) as long as the brushing protocol continued. Post-brushing fluoride release after week 1 was lower than the baseline values for all groups.

**Figure 3:**
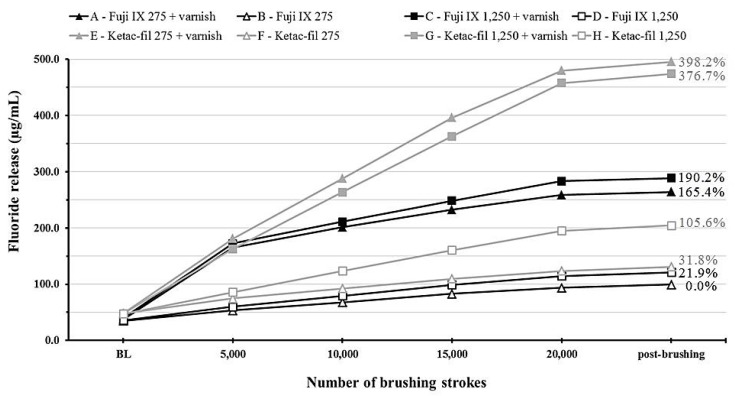
A line graph showing cumulative fluoride release from the experimental groups at different time points throughout the study period. Values on the right indicate percentage increases in cumulative fluoride release compared to group B.

Time–varnish and time–material interactions were significant (*p* < 0.001) indicating a large increase in fluoride release depending on the material (higher for KF). This is illustrated clearly in the cumulative fluoride release data (Figure 3) which show that the varnish- and material-induced effects were dependent on the material and the time point. 

Group B was considered as the basis for cumulative fluoride release since it had the lowest values. The highest percentage increases were 190.2% (group C) and an almost 4-fold increase (group E) for F9 and KF, respectively. 

## 4. Discussion

Glass ionomer cement (GIC) restorations are still considered among the modalities for increasing the fluoride content within the oral cavity leading to reduced carious incidence in cases of severe caries disease [16,17,18]. It is known that brushing GIC restorations will lead to an enhanced fluoride release [11]. Further, brushing with slurries that contain high abrasive content can lead to further fluoride release enhancement [12]. This is particularly helpful in patients with multiple carious lesions; however, caution must be taken since carious lesions tend to have a softer surface layer compared to sound enamel probably leading to more surface abrasions of these lesions [19,20]. Nonetheless, GIC restorations are used as a caries-control intermediary step in cases with high caries incidence or rampant caries. This would allow fluoride to be available intraorally in saliva, oral fluids, and mucosa in order to boost remineralization and decrease demineralization [21,22].

Our objective was to study the effects of slurry fluoride concentration and material brand on the fluoride release from conventional glass ionomer cements in the presence of a fluoride varnish-simulated step. As hypothesized, a marked increase in fluoride release was associated with the simulated varnish step regardless of material type. In addition, the material effect was observed at baseline with higher fluoride release values with KF as well as high microhardness figures. Even though the surface abrasion was less in KF owing to higher VHN numbers, the increase in fluoride release was more sustained than F9.

The effect of slurry fluoride concentration on the fluoride release was less straightforward. When each material was considered separately, the effect was non-significant (*p* = 0.259 for F9 and *p* = 0.054 for KF). However, when the effect of the slurry was collectively considered, a significant effect was found (*p* = 0.032). This would probably indicate that the effect of the slurry fluoride concentration is dependent on the material. This conclusion is visualized in Figure 2, which illustrates that the overlap between standard error bars for the non-varnish KF groups (F and G) is minimal. Nevertheless, the effect of the 1250 ppm slurry appears minute in the varnish KF groups as well as the F9 groups. According to Staun Larsen and collaborators [23], the fluoride concentration in the saliva and oral mucosa was dependent on the fluoride concentration of the toothpaste. Even though GIC is a different substrate, our values for the KF go well with their finding.

Conventional GIC materials were used in the present study rather than the mechanically superior resin-modified glass ionomer formulations because the conventional GIC provides more fluoride release comparatively [8]. These restorations can be used as interim restorations to control dental caries. Thus, they are potentially subjected to brushing abrasion for prolonged periods. Mechanical properties tend to materialize after the initial maturation stage which is within 24 h for conventional GIC [24]. Moreover, this period also reflects the highest fluoride release values [7,25,26,27].

The recommended duration of a brushing session is 2 min; this would translate into 20 s per sextant and approximately 5 s per surface [28]. Previously, it was reported that this would translate into 15 strokes per brushing session resulting in approximately 5000 brushing strokes needed to simulate 6 months of brushing [29]. Consequently, specimens in the current investigation were subjected to a simulated abrasion equal to 2 years (total of 20,000 strokes) of brushing.

Commonly recommended brushing protocols for patients involve two brushing sessions that can be increased to three daily sessions for high and extremely high caries-risk individuals [13,15]. This could potentially lead to more surface abrasion of the glass ionomer restorations since these restorations tend to be softer than other restorative formulations [30]. Based on results from the current study, F9 is more prone to this accelerated abrasion effect without the added benefit of increasing the fluoride release.

Post-brushing, only KF groups subjected to the varnish step had more fluoride release when compared to the other groups. The added effect of the fluoride varnish step was clearly seen for KF compared to F9 possibly indicating a better potential for fluoride recharge and rerelease. Even though KF was relatively harder, indicating more resistance to surface abrasion, fluoride release was higher possibly owing to a better recharging potential. It was shown previously that GICs with high fluoride release had more particles exposed owing to the vulnerability to surface abrasion which was not the case in the current investigation since KF had higher microhardness values [12,31].

The fluoride recharging of GIC depends on multiple factors including type of the material, concentration of fluoride in the recharging agent, and the frequency of exposure [32,33]. It was shown previously that a higher concentration of the surrounding medium leads to higher recharging of GIC. The fluoride rerelease could be due to the washing of fluoride ions from the glass matrix or from the material’s surface [34]. Overall, GIC has a good capability to accept and release fluoride due to the loosely bound water that can be exchanged with the surrounding medium via passive diffusion [35,36]. According to previous reports, materials with high initial fluoride release tend to show higher affinity to fluoride as well as a higher recharging potential [27,35,37,38]. This is in agreement with our findings in which KF showed higher initial fluoride release and was associated with more sustained fluoride rerelease.

Abrasion is a time-dependent process that will increase with each abrasive episode [20,39,40]. This goes well with our observations that specimens were more abraded as the brushing protocol continued. We witnessed the same phenomenon in our previous investigation where abrasion was accompanied by more fluoride release in the brushed groups [12]. Still, the initial high fluoride values diminished with each day of the protocol. This is in agreement with previous investigations that reported high fluoride release within the first two days which tapered down markedly to reach an ambient sustained fluoride release over multiple weeks [41,42].

Choosing a restorative material that releases significant fluoride in the oral cavity is very important, especially in high-risk patients. However, other material properties, such as mechanical characteristics and resistance to abrasion, must also be considered when choosing the brand and the type of GIC to be placed.

Cumulative fluoride release values show a clear difference between the two tested materials at the end of the simulated 2-year brushing duration. This finding along with the microhardness results necessitate careful selection of glass ionomer formulations based on the patient’s caries risk and oral hygiene habits. Further, the application of a fluoride varnish would allow faster recharging of these GIC restorations with more subsequent release that could tip the caries balance towards remineralization if other caries-control modalities are implemented.

Increasing fluoride concentrations has been linked previously with decreasing the risk of dental caries. Although the caries process involves many other factors including frequency of sugar consumption, salivary flow rate, and oral hygiene practices, still, fluoride is considered as a strong modulating factor that can affect the association of caries-relevant elements. The boost in fluoride release observed in the KF groups that were exposed to a high-concentration solution could be very relevant in providing additional free fluoride ions that could enhance remineralization of incipient lesions and decrease the demineralization of susceptible tooth structures. Increasing the exposure to fluoride via oral health care products is included in all evidence-based caries management protocols and clinicians should consider it as an integral component of their caries preventive/management protocol. 

As is the case with other in vitro investigations, this study has some limitations. Accelerated simulated abrasion does not entirely mimic oral conditions, especially as the exposure to dietary sources of fluoride or thermocycling episodes is concerned. Further, DI water was used as the storage medium instead of saliva due to infection control hazards. Fluoride release in saliva tends to be reduced compared to water indicating that released fluoride values in vivo would be lower (approximately 17–25%) compared to figures recorded in the present investigation [5,43]. Still, the insertion of GIC was associated with an increase of salivary fluoride levels [44,45].

## 5. Conclusions

In conclusion, conventional glass ionomer materials subjected to brushing and immersion in a 22,500 ppm F solution led to high fluoride release that was more sustained for KF compared to F9. Fluoride content in the brushing slurry did not provide additional effect on fluoride release after 20,000 strokes of brushing. Material type was associated with different baseline fluoride release values as well as different fluoride release sustainability potential.

## Figures and Tables

**Table 1:** Summary of the experimental groups used in the study with their respective treatments.

Group ^1^	Material	Slurry Fluoride Content (ppm F as NaF) ^2^	Fluoride Varnish Step ^3^
A	GC Fuji IX	275	Yes
B		275	No
C		1250	Yes
D		1250	No
E	3M ESPE Ketac-fil	275	Yes
F		275	No
G		1250	Yes
H		1250	No

^1^*n* = 6 per group. ^2^ all slurries contained 2.5 g carboxymethylcellulose Blanose 7MF (Ashland, Inc., Covington, OH, USA), 5 g glycerol Zeodent 113 precipitated silica abrasives (J.M. Huber Corporation, Edison, NJ, USA; relative enamel abrasivity = 4.01 ± 0.28) and were made up to 60 g using deionized water. ^3^ equivalent to 22,500 ppm F as NaF.

**Table 2:** Mean and standard deviation of fluoride release (µg/mL) among the different groups at different time points.

Time Point	Varnish Step	Slurry Fluoride Content (ppm)			
		275		1250	
		Fuji IX (F9)	Ketac-fil (KF)	Fuji IX (F9)	Ketac-fil (KF)
Baseline	Yes	37.4 ± 9.6 ^A/a^	47.9 ± 16.5 ^A/a^	39.6 ± 18.1 ^A/a^	47.7 ± 13.7 ^A/a^
	No	35.2 ± 12.1 ^A/a^	48.1 ± 12.7 ^A/a^	35.8 ± 18.7 ^A/a^	47.3 ± 6.1 ^A/a^
5000 strokes	Yes	128.3 ± 11.0 ^A/a^	133.0 ± 29.4 ^A/a^	133.0 ± 46.7 ^A/a^	114.9 ± 17.8 ^A/a^
	No	18.3 ± 7.7 ^A/b^	26.7 ± 9.6 ^A/b^	24.3 ± 15.7 ^A/b^	38.6 ± 12.5 ^A/b^
10,000 strokes	Yes	35.6 ± 4.9 ^A/a^	106.8 ± 31.6 ^B/a^	38.2 ± 9.7 ^A/a^	100.9 ± 13.5 ^B/a^
	No	14.2 ± 6.4 ^A/b^	17.5 ± 5.2 ^A/b^	19.3 ± 11.0 ^A/b^	37.7 ± 8.2 ^A/b^
15,000 strokes	Yes	31.2 ± 4.2 ^A/a^	108.2 ± 26.7 ^B/a^	37.6 ± 6.4 ^A/a^	99.6 ± 13.3 ^B/a^
	No	15.3 ± 6.0 ^A/b^	17.3 ± 6.3 ^A/b^	19.7 ± 12.6 ^A/a^	36.8 ± 11.1 ^A/b^
20,000 strokes	Yes	26.2 ± 3.9 ^A/a^	83.6 ± 24.7 ^B/a^	34.7 ± 8.7 ^A/a^	94.7 ± 23.2 ^B/a^
	No	10.8 ± 3.9 ^A/b^	14.1 ± 4.0 ^A/b^	15.6 ± 8.7 ^A/a^	34.7 ± 10.2 ^A/b^
1 week post-brushing	Yes	5.1 ± 1.3 ^A/a^	15.7 ± 8.6 ^B/a^	5.2 ± 1.4 ^A/a^	16.1 ± 7.6 ^B/a^
	No	5.6 ± 2.3 ^A/a^	7.3 ± 2.6 ^A/a^	6.6 ± 3.6 ^A/a^	9.2 ± 2.9 ^A/a^

Same uppercase superscripts indicate non-significant differences (*p* > 0.05) between F9 and KF at the same fluoride and varnish parameters. Same lowercase superscripts indicate non-significant differences (*p* > 0.05) between varnish and non-varnish groups within the same material and fluoride levels. Effect of slurry fluoride level was not significantly different (*p* > 0.05) for either material at all time points.
